# Analysis of Genome-Wide Changes in the Translatome of Arabidopsis Seedlings Subjected to Heat Stress

**DOI:** 10.1371/journal.pone.0071425

**Published:** 2013-08-19

**Authors:** Emilio Yángüez, Ana B. Castro-Sanz, Nuria Fernández-Bautista, Juan C. Oliveros, M. Mar Castellano

**Affiliations:** 1 Centro de Biotecnología y Genómica de Plantas, INIA-UPM, Madrid, Spain; 2 Centro Nacional de Biotecnología-CSIC, Madrid, Spain; National Institute of Genomic Medicine, Mexico

## Abstract

Heat stress is one of the most prominent and deleterious environmental threats affecting plant growth and development. Upon high temperatures, plants launch specialized gene expression programs that promote stress protection and survival. These programs involve global and specific changes at the transcriptional and translational levels. However, the coordination of these processes and their specific role in the establishment of the heat stress response is not fully elucidated. We have carried out a genome-wide analysis to monitor the changes in the translation efficiency of individual mRNAs of *Arabidopsis thaliana* seedlings after the exposure to a heat shock stress. Our results demonstrate that translation exerts a wide but dual regulation of gene expression. For the majority of mRNAs, translation is severely repressed, causing a decreased of 50% in the association of the bulk of mRNAs to polysomes. However, some relevant mRNAs involved in different aspects of homeostasis maintenance follow a differential pattern of translation. Sequence analyses of the differentially translated mRNAs unravels that some features, such as the 5′UTR G+C content and the cDNA length, may take part in the discrimination mechanisms for mRNA polysome loading. Among the differentially translated genes, master regulators of the stress response stand out, highlighting the main role of translation in the early establishment of the physiological response of plants to elevated temperatures.

## Introduction

Because of their immobile nature, plants have adopted versatile strategies to grow and propagate under rapidly changing environmental challenges, such as low or high temperatures, high salt soils or drought. These strategies involve deep molecular changes covering almost every step of gene expression: from transcription to mRNA processing, translation or protein turnover [1], [2], [3], [4]. Although the importance of all these processes in the modulation of the response to stress has been widely accepted, our knowledge of the regulation of each individual step is far from being balanced. For years, the study of gene expression under stress has been focused, almost exclusively, on the transcriptional level while the relevance of all the post-transcriptional regulatory mechanisms has been underestimated and restricted to a few examples [5]. In that sense, despite several studies demonstrate that general translation inhibition and selective translation of some mRNAs are key points in the plant adaptation process to different abiotic threats, including hypoxia [6], [7], [8], light [9], [10], sublethal cadmium intoxication [11], dehydration [12], [13], sucrose starvation [14] and saline stress [15], the mechanisms involved in this regulation are far from being understood [16]. Nevertheless, the comprehensive analysis of all these data clearly establishes that, in general terms, translational modulation differs markedly depending on the stress and its characteristics. This situation is even more complex when assessing a whole plant, as translation is also differentially regulated depending on the cell type [8].

Temperature is one of the most limiting environmental factors affecting life on Earth and it is especially relevant for sessile organisms as plants. Indeed, high temperature is considered one of the most deleterious stresses for plants, as it adversely impacts almost all aspects of plant development, including growth, reproduction and yield [1], [17], [18]. Upon heat stress, plants reprogram their gene expression in an attempt to cope and prevent the damage caused by high temperatures. This deep reprogramming involves a wide regulation of transcription that affects a relevant part of the transcriptome [19], [20], [21], [22]. Among the upregulated genes, those coding for heat shock proteins, the molecular chaperones that prevent protein misfolding and aggregation, clearly stand out [23], [24]. The rest of the transcriptionally regulated genes cover multiple functions, such as transcription, translation, signaling, metabolism and general stress response. However, this mRNA steady-state scenario may not reflect the protein level output, since, after being transcribed, the mRNAs should be translated and translation is also widely altered in these conditions [25], [26], [27], [28]. In this regard, recent analyses done in Arabidopsis and *O. sativa* cultured cells have pointed out that, upon short heat stress treatments, translation is generally and specifically regulated [27], [28]. However, heat is a complex threat that does not affect all cells within plant organs in a uniform way. Indeed, the effect of high temperatures on plant cells differs markedly depending on the stage of growth and the type of plant tissue, questioning the possible extrapolation of the data obtained in cultured cells to the real response in whole plants. In addition, certain stages of the plant cycle, as it is the case of seedlings, are more susceptible to heat than others [29]. Thus, heat stress studies during the especially vulnerable growth stages may unravel novel differentially regulated proteins with critical functions in plant stress adaptation and survival. Despite their relevance, these additional physiological studies are still lacking. In addition, the mechanisms involved in the preferential translation of mRNAs in response to heat in plants remain uncovered.

In this report, we have carried out a genome-wide analysis to monitor the changes in the translational profiling of *Arabidopsis thaliana* seedlings after the exposure to a heat shock stress. This study has allowed to evaluate, for the first time, the specific contribution of translation to the regulation of gene expression in response to high temperatures in a whole plant organism. Our results point out that, superimposed to transcriptional changes, translation constitutes an important layer for the regulation of gene expression during heat stress. Although translation widely represses general gene expression under heat shock, this repression is selectively modulated for specific subsets of mRNAs. Thus, some mRNAs coding for relevant proteins involved in the general stress response are more recalcitrant to translation inhibition, while mRNAs coding for proteins related with translation and ribosome biogenesis are more sensitive to the global inhibition of translation. Among the mRNAs preferentially translated upon heat stress, well characterized transcriptional regulators as STZ/ZAT10 and DREB2B stand out. These differentially translated regulators modulate the expression of downstream genes involved in drought and salt-stress response in *Arabidopsis thaliana*, contributing, in such a way, to enhance salinity, drought and heat stress tolerance in plants [30], [31]. As differential translation selectively affects the levels of proteins that are relevant to cope with the stress and of proteins involved in highly energy-demanding processes, these data reflect the key role of translation in the successful establishment of the thermotolerance response. Analyses of the sequences of the differentially translated mRNAs unravel that some features, like the 5′UTR G+C content and the cDNA length, may take part in the discrimination mechanisms that control mRNA polysome loading upon high temperatures. The identification of these features significantly contributes not only to our current understanding of how plants adapt to this harmful environmental stress, but it also may help to provide insights into the still undiscovered mechanisms that promote translational inhibition under high temperatures. Furthermore, this knowledge may open up new strategies for the generation of powerful genetic tools to improve crop thermotolerance by promoting the expression of high levels of ectopic protective proteins in a situation in which, otherwise, would be translationally repressed.

## Materials and Methods

### Plant material and growth conditions

*Arabidopsis thaliana* ecotype Columbia-0 seeds were surface sterilized using 0.05% Tween-20 and ethanol for 5 min, sown on MS medium plates and kept at 4°C in darkness for 2 days for stratification. Plates were vertically oriented in a growth chamber under a 16 h light/8 h dark cycle for 7 days at 22°C.

### Metabolic labeling of newly synthesized proteins

For the continuous labeling of newly synthesized proteins, 7-day-old Arabidopsis seedling were incubated, at the indicated times and temperatures, with liquid MS media supplemented with 50 µCi/ml ^35^S-methionine/^35^S-cysteine (EasyTag™ EXPRESS^35^S Protein Labeling Mix, Perkin Elmer) for 15 minutes. Seedlings were washed three times with liquid MS, quickly frozen in liquid nitrogen, ground to powder and solubilized in Laemmli sample buffer. Equivalent amounts of plant extracts were subjected to 12.5% SDS-PAGE electrophoresis and ^35^S-labeled proteins were detected by autoradiography.

### Isolation and quantitation of total and polysomal RNA

**Figure 1:**
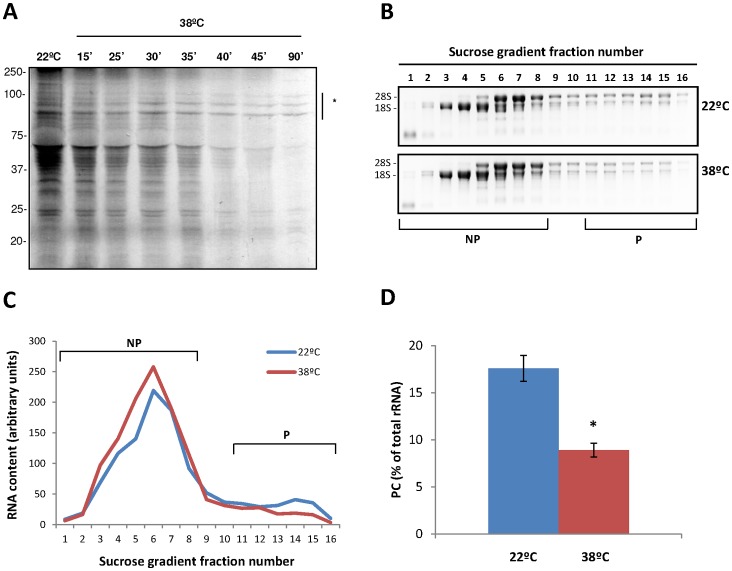
Heat triggers a general inhibition of protein synthesis. (**A**) Metabolic labeling of *de novo* synthesized proteins from Arabidopsis seedlings grown at 22°C or after a heat shock of 38°C during the indicated times. High molecular weight heat-shock proteins are indicated by an asterisk. (**B**) Verification of the quality of the polysome preparation. Equal volumes of the last 16 fractions of the sucrose gradient from control or heat-stressed Arabidopsis seedlings were subjected to electrophoretic analysis. According to the distribution of 18 S and 28 S rRNAs, fractions 1–8 were designated as non-polysomal (NP), while fractions 11–16 were considered as polysomal (HP) fractions. (**C**) Quantification and graphic representation of the polysome profiles from the samples in (B). (**D**) Polysome content (PC) of control or heat-stressed Arabidopsis seedlings was calculated as described in materials and methods. Values are mean percentages from three biological samples (*P<0.05).

Seven-day-old seedlings were subjected to heat stress by incubation at 38°C for 45 min. For the control conditions, plates taken directly from the growth chamber were used. Seedlings were quickly frozen in liquid nitrogen, ground to powder and stored at −80°C. Polysomes isolation by differential centrifugation was performed as described in [12] with slight modifications. Briefly, 2 g of frozen tissue was thawed in 2 ml of polysome extraction buffer (200 mM Tris (pH 9.0), 200 mM KCl, 26 mM MgCl_2_, 25 mM EGTA, 100 µM 2-mercaptoethanol, 50 µg/ml cycloheximide, 50 µg/ml chloramphenicol, 1% (v/v) Triton X-100, 1% (v/v) Tween-20, 1% (v/v) NP-40, 2% (v/v) polyoxyethylene-10-tridecyl-ether, 1% (v/v) deoxycholic acid). The crude cell extract was clarified by centrifugation at 13,500 g for 15 min at 4°C and the OD_260_ of the supernatant determined. 7000 units (OD_260_) were layered on top of a 10 ml 15–40% (w/v) sucrose gradient and centrifuged at 38000 rpm (178,000 g) for 3 h 40 min at 4°C (SW41Ti rotor in a Beckman L-100XP ultracentrifuge). Twenty fractions of 600 µl were manually collected and RNA extraction was performed using TRIzol reagent (Invitrogen) according to manufacturer's protocol. The polysome profile was determined by measuring the RNA content of each fraction at OD_260_. The quality of the polysome preparation was evaluated by electrophoretic analysis of rRNA distribution in a 1.5% agarose gel containing formaldehyde. In the case of the figures 1B–C, only the data for the last 16 fractions of the gradient are shown. Quantitative polysome gradient fractionation was performed using three independent biological samples. Polysome content (PC) of control or heat-stressed Arabidopsis seedlings was used as a quantitative indicator of the translational state [7], [12], [13]. Briefly, total or polysomal rRNA contents were estimated by calculating the area under the whole polysome profile or under the polysomal fraction curves (11–16), respectively, after subtracting the gradient baseline. PC was expressed as the percentage of total rRNA in polysomal fractions (rRNA_P_/rRNA_TOT_*100) in each sample. The calculated PC was used as a correction factor for polysomal RNA samples in microarray hybridization and qRT-PCR analysis.

For polysome-bound (PB) RNA extraction, fractions 11–16 were pooled and the RNA was precipitated with LiCl overnight at −20°C and subsequently purified using RNeasy Minielute Cleanup Kit (Qiagen), according to manufacturer's protocol. This step was critical to eliminate any rest of heparin that could inhibit the retrotranscription during the labeling process [32]. Total RNA from the same biological samples was isolated from crude cell extract in polysome extraction buffer prior to the loading of the gradient and extracted using TRIzol reagent (Invitrogen) according to manufacturer's protocol, precipitated with LiCl overnight at −20°C and subsequently purified using RNeasy Minielute Cleanup Kit (Qiagen), according to manufacturer's protocol. The amount and quality of the RNA preparations was evaluated by electrophoretic analysis and using the Agilent's 2100 Bioanalyzer.

### Affimetrix GeneChip hybridization and data analyses

Microarray analyses were performed to quantify individual changes in total mRNA and in polysome-bound mRNA from control (mRNA_TOT,22_ and mRNA_PB,22_) and heat-stressed plants (mRNA_TOT,38_ and mRNA_PB,38_). Two biological replicates for each sample were independently hybridized to ATH-1 microarrays (Affymetrix), containing 22500 transcript variants from 24000 Arabidopsis genes. Each sample was added to a hybridization solution containing 100 mM 2-(N-morpholino) ethanesulfonic acid, 1 M Na+, and 20 mM of EDTA in the presence of 0.01% Tween-20. Hybridization was performed for 16 h at 45°C. Each microarray was washed and stained with streptavidin–phycoerythrin in a Fluidics station 450 (Affymetrix) and scanned at 2.5-μm resolution in a GeneChip® Scanner 3000 7 G system (Affymetrix). Data analyses were performed using GeneChip® operating software (GCOS) to generate the corresponding CEL files. Raw intensities were summarized by Robust Multi-Array (RMA) method [33]. To quantify the amount of individual mRNAs in the polysomal samples, the RMA data values of mRNA_PB,38_ samples were further adjusted based on the polysome content (PC), as previously described [7]. Briefly, the normalization factor was generated from the quantitative measurement of the relative polysome content (PC_38°C_/PC_22°C_) in each biological replicate (0.558 and 0.538, respectively). Initially, individual changes in total mRNA (ΔmRNA_TOT_) and polysome binding (ΔmRNA_PB_) were evaluated. After data normalization and adjustment, each probe was tested for statistically significant changes in expression using an empirical Bayes moderated t statistic [7], [34]. To control the false discovery rate (FDR), p-values were corrected using the method of Benjamini and Hochberg [35]. For all comparisons, probes with a ≥2-fold variation and a FDR <0.05 were considered as significantly changed. Translation efficiency at 22°C or 38°C was defined as the ratio between mRNA abundance in polysomal fractions and in total mRNA (mRNA_PB,22°C_/mRNA_TOT,22°C_ and mRNA_PB,38°C_/mRNA_TOT,38°C,_ respectively). To estimate the relative translation efficiencies for each mRNA in response to heat stress (ΔmRNA_PB_/ΔmRNA_TOT_), changes in polysomal association (mRNA_PB,38°C_/mRNA_PB,22°C_) were corrected by the corresponding variations in steady-state mRNA levels (mRNA_TOT,38°C_/mRNA_TOT,22°C_). All statistical analyses were performed with the packages “affy” and “limma” from Bioconductor (http://www.bioconductor.org). FIESTA system was used to visualize the results [36].

Both raw and normalized microarray data are deposited in Gene Expression Omnibus database (GEO-NCBI) (http://www.ncbi.nlm.nih.gov/geo/) with accession code: GSE44053. The following link has been created to allow review of record GSE44053 while it remains in private status: http://www.ncbi.nlm.nih.gov/geo/query/acc.cgi?token=fdobdkgcyceginw&acc=GSE44053.

### Gene onthology and mRNA feature analyses

Functional analysis of translational categories was carried out by single or set enrichment using GeneCodis [37], [38], [39] and FatiScan (Babelomics 4.3 suite) [40], respectively. Only specifically enriched GO “biological processes” in both analyses were considered. Data sets for the 5′-UTR, 3′-UTR, CDS and cDNA sequences were retrieved from The Arabidopsis Information Resources (TAIR) (ftp://ftp.arabidopsis.org/home/tair/Genes/TAIR10_genome_release). A database including all the putative Arabidopsis uATG was kindly provided by Albrecht Vonarnim (University of Tenesse). The representative gene model was used as the reference gene model for the analysis. Only genes with complete information for the different features analyzed were considered (16098). The minimum energy folding for the different 5′-UTR and 3′-UTR sequences was calculated using UNAFold3.8 (hybrid-ss-min -E script) running on Windows 7 [41]. Basic and advanced statistical analyses were performed in Excel 2012 and IBM SPSS Statistics 20, respectively. The search for overrepresented cis-elements in the different mRNA classes was performed using Multiple Em for Motif Elicitacion (MEME-Chip) [42]. The occurrence of the identified overrepresented elements in the rest of the groups was evaluated using Find Individual Motif Occurences (FIMO) [43].

### qRT-PCR analyses

For the confirmation of the heat induced changes in the ATH-1 Hybridization arrays, three independent biological replicates were performed for each group of samples. For total and polysomal RNA, the complementary DNA (cDNA) synthesis was performed using a fixed amount of RNA (1 µg per sample) with the High Capacity RNA-to-cDNA Kit (Invitrogen) according to manufacturer's protocol. qRT-PCRs were performed in 10 µl reactions using Kapa Sybr Fast qPCR Kit (Kapabiosystems) in an Illumina Eco Real PCR System thermocycler, using the primers listed in Table S1. The 18S rRNA was used as control for normalization of the amount of RNA. As a fixed amount of RNA was used for the reverse transcription, a correction to account for the reduction of mRNA_PB,38°C_ induced by the stress was done. Briefly, polysomal content (PC) was determined independently for each biological triplicate as above explained. To obtain the final estimation of the mRNA_PB,38_, the values obtained by qRT-PCR for the different mRNAs in mRNA_PB,38_ samples were multiplied by the relative PC of each biological replicate.

For the analysis of mRNA distribution through the different fractions of the sucrose gradient, three independent biological replicates were performed. In this particular case, the original 20 fractions of the gradient were pooled in 10 fractions of 1.2 ml. The RNA in each sample was subsequently purified using TRIzol reagent (Invitrogen), followed by RNeasy Minielute Cleanup Kit (Qiagen), according to manufacturers' protocol. In this particular case, the complementary DNA (cDNA) synthesis was performed as above described but using a fixed volume of the RNA sample from each fraction. qRT-PCRs were performed as above described. The relative amount of mRNA in each fraction was estimated from the Ct value and expressed as a percentage of the total mRNA in the gradient.

## Results

### Heat stress dynamically regulates protein synthesis and polysome abundance

In order to investigate the global effect of heat stress on plant translation and to establish the experimental conditions, 7-day-old Arabidopsis seedlings were incubated at 38°C for the different times indicated in Figure 1A and the incorporation of ^35^S-methionine in the *de novo* synthesized proteins was monitored. As previously described [25], [44], the incorporation of ^35^S-methionine was clearly decreased upon heat treatment, pointing out the global inhibition of protein synthesis in response to the stress. The severity of this response was dependent on the stress duration, since, after 90 minutes of heat exposure, global protein synthesis was almost completely inhibited. This general inhibition was concomitant to the selective synthesis of heat shock proteins (HSPs). Subsequent experiments were performed with 45 minutes of heat treatment at 38°C as, at this time, the effect of the stress in general translation inhibition and in the further accumulation of HSPs is clearly detectable, indicating that at that point the plant response to the heat is properly established.

Polysome profiling allows the separation of mRNAs in a sucrose gradient according to the number of ribosomes they are associated with, reflecting their translational status. Thus, the mRNAs in the non-polysomal fraction (NP) are translationally inactive or repressed, while the mRNAs in polysomal fractions (P) are actively translated [8]. Therefore, this technique could be used to uncover changes in the translatability of the mRNAs under different conditions. To deeply characterize the plant translational response to high temperatures, control or heat-stressed seedlings were used for polysome profiling analysis, and their relative polysome content was evaluated. A representative example of the distribution of rRNAs in control and heat-stressed seedlings over the different fractions of the sucrose gradient is shown in Figure 1B, and the quantification and graphic representation of the polysome profiles from the same samples is shown in Figure 1C. Fractions 1–8 were designated as non-polysomal fraction (NP), while fractions 11–16 were considered as polysomal (P) fractions. As observed in these figures, the amount of polysomes in P fractions is clearly reduced under the tested stress conditions, further confirming that general translation is inhibited in response to heat stress. This reduction in the amount of polysomes was accompanied by an increase in the 80 S monoribosome and free ribosomal subunits (fractions 3–7), which is characteristic of an impairment of the translation initiation.

A more quantitative indicator of the translational activity was obtained by calculation of the polysome content (PC). PC is the percentage of total rRNA in polysomal fractions (rRNA_P_/rRNA_TOT_) and is calculated as indicated in Materials and Methods. In control seedlings, almost 20% of the ribosomal subunits are included in polysomes, whereas heat stress reduced the PC to less than 10% (Figure 1D). All together, these results indicate that global translation is reduced by a 50% during the response to heat stress in Arabidopsis and that this reduction is likely related with a defect in the translation initiation.

### Heat stress induces rapid alterations in the translational status of individual mRNAs

**Figure 2:**
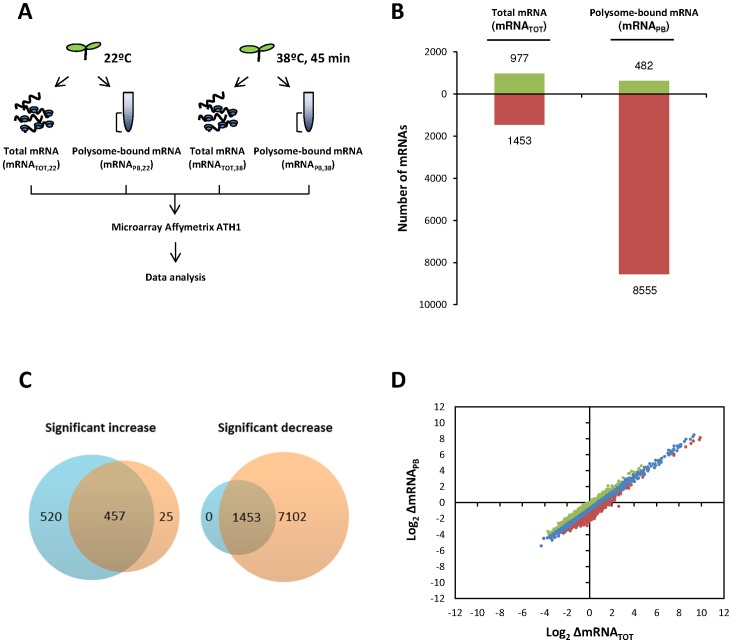
Comparison of heat-induced changes in steady-state and polysome bound mRNAs from Arabidopsis seedlings. (**A**) Illustration of the experimental design. Total and polysome-bound mRNA from control or heat-stressed seedlings were isolated in parallel for microarray hybridization to Arabidopsis whole-genome Affymetrix ATH-1 platform and subsequently analyzed. (B) Graphic representation of the number of mRNAs with ≥2-fold variations in steady-state levels (mRNA_TOT_) or in polysome association (mRNA_PB_) in response to the heat treatment. Green and red colors represent upregulated and downregulated mRNAs under heat stress, respectively. (**C**) Venn diagrams of heat-stress upregulated and downregulated mRNAs. mRNAs that only experienced significant variations in steady-state levels (≥2-fold variations in mRNA_TOT_, FDR<0.05) are shown in blue, those that only experienced significant changes in their association to polysomes (≥2-fold variations mRNA_PB_, FDR<0.05) are shown in red. Merged section included those transcripts that significantly changed at both, transcriptional and translational, levels (≥2-fold variations in mRNA_TOT_ and mRNA_PB_, FDR<0.05) in response to the heat treatment. (**D**) Genome-wide comparison of the heat-induced changes in the transcriptome (x-axis, log_2_ΔmRNA_TOT_) and the translatome (y-axis, log_2_ΔmRNA_PB_). The log_2-_values of the fold changes were plotted for all the probe sets included in the Affimetrix ATH-1 microarray (n = 22810). mRNAs with an average translation efficiency (log_2_ΔmRNA_PB_/ΔmRNA_TOT_) are shown in blue, while mRNAs with ≥1.5 or ≤1.5 fold the average translation efficiency are shown in green and red, respectively.

Although general translation is decreased in response to high temperatures in Arabidopsis, relative variations in the translation efficiency of the different mRNAs could significantly alter the accumulation of the encoded proteins and regulate the stress response. In addition, changes in the association of mRNAs to polysomes under different conditions could reflect differences in total mRNA abundance due to transcriptional regulation. To evaluate the relevance of the translational regulation in response to high temperatures at the individual mRNA level and to rule out the contribution of transcriptional changes to the association of the individual mRNAs to polysomes, microarray hybridization of total and polysome-bound mRNAs was performed using Arabidopsis whole-genome Affymetrix ATH-1 platform. This system allows controlling the expression of 22810 probe sets, which correspond with approximately 24000 genes. The experimental design for obtaining the biological replicates is illustrated in Figure 2A. The relative amount of each transcript in the different samples was first evaluated using FIESTA interactive server [36]. With an arbitrary two-fold cut-off (FDR<0.05), 977 mRNAs increased their abundance in response to heat, whereas only 482 mRNAs were found to be more associated to polysomes (Figure 2B). On the other hand, 1453 mRNAs decreased their total mRNA level, while 8555 were less loaded in polysomes upon heat treatment. Venn diagrams were used to further quantify these data (Figure 2C). 457 mRNAs were found to be up-regulated both in their steady-state levels and association to polysomes in response to heat, while the abundance of 520 and 25 transcripts was found to be increased only in mRNA_TOT_ (blue) or mRNA_PB_ (red), respectively. Regarding down-regulated transcripts, 1453 mRNAs were found to be co-ordinately down-regulated, while 7102 mRNAs were only differentially regulated at the translational level. These data clearly establish that the mRNA association to polysomes is mainly repressed in response to heat stress. Moreover, these findings point out the relevance of the translational regulation in response to high temperatures, as the number of mRNAs that are distinctly associated to polysomes is clearly higher than the number of transcripts with significant variations in their steady-state abundance. Altogether, these data suggest that translation constitutes an important layer for the regulation of gene expression during heat stress conditions.

For a more detailed analysis individual changes in total mRNA in response to heat (ΔmRNA_TOT_) were plotted, as log_2_-transformed values, against the heat induced mRNA variations in polysome binding (ΔmRNA_PB_) (Figure 2D). After 45 minutes at 38°C, broad changes in both transcription and association to polysomes were detected in a range of more than 500-fold (∼2^9^), demonstrating that both processes play an important role in the heat stress regulation of gene expression. For the majority of messengers (blue), heat induced a decrease of 50% in average in their association to polysomes (Log_2_ΔmRNA_PB_). Although decreased, the change in polysome binding in this group tightly correlates with the variations in total mRNA (Log_2_ΔmRNA_TOT_). In contrast, for a relevant group of transcripts (1389 genes covering the 8.6% of the analyzed genes), variations in polysome association is not as directly determined by changes in steady-state levels (Figure 2D dots in green and red), indicating that their translation regulation in response to heat should be more complex.

**Figure 3:**
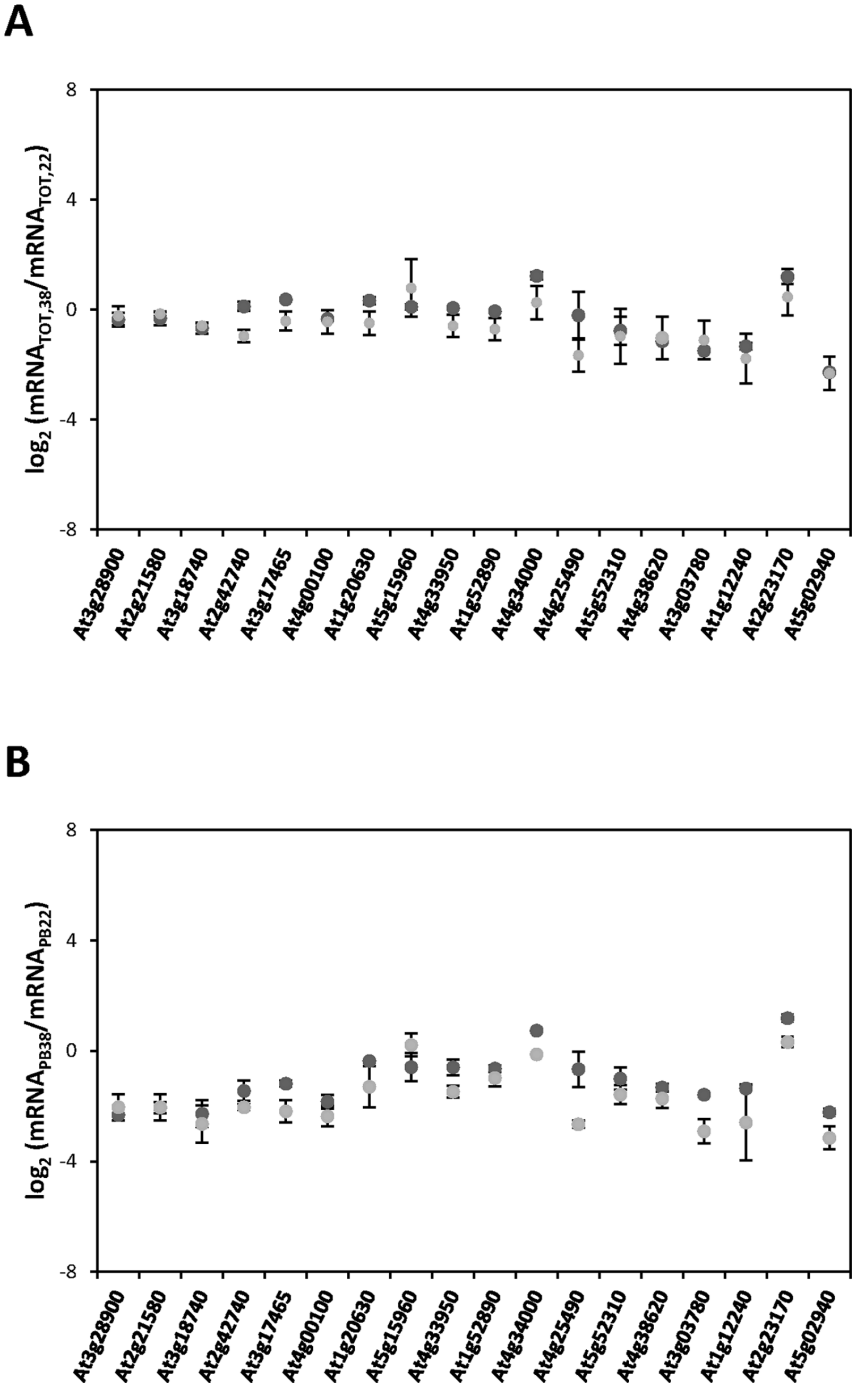
Confirmation of the heat-induced changes in transcriptome and translatome observed in the ATH-1 hybridization arrays by qRT–PCR. For a representative group of mRNAs, the log_2_ changes in response to heat stress in steady-state levels (**A**) and in polysome-bound abundance (**B**) obtained from the ATH-1 hybridization arrays (dark-grey circles) were compared with the corresponding data obtained by the analysis of three independent biological replicates by qRT-PCR (light-grey circles). Mean (points) and standard deviations from the different replicates are shown.

**Figure 4:**
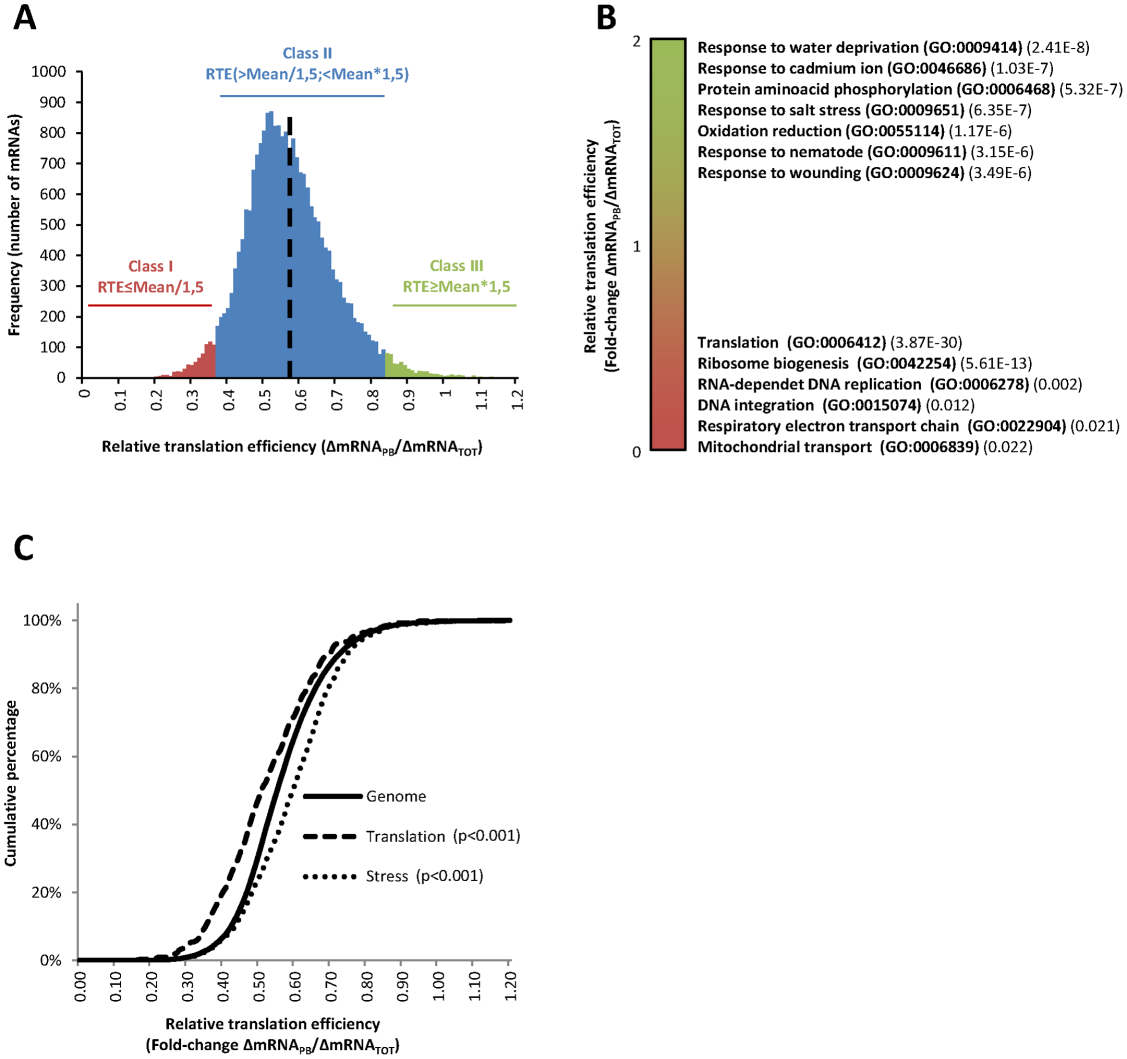
Heat selectively regulates translation of functionally relevant cohorts of mRNAs. (**A**) Density distribution of the relative translation efficiencies (ΔmRNA_PB_/ΔmRNA_TOT_) of the mRNAs selected for the bioinformatics analysis (n = 16098) in response to heat-stress. The average relative translation efficiency of the distribution (0.564) is shown as a dotted black line. The categories, in which mRNAs were classified for subsequent analysis, are also shown in the figure. Class I (red; n = 696) and Class III (green; n = 456) included mRNAs with a relative translation efficiency ≤1.5 fold or ≥1.5 fold the average translation efficiency of the distribution, respectively. Class II (blue; n = 14946) included the rest of the genes within the described parameters. (**B**) Functional analysis of the selected mRNAs according to their relative translation efficiencies using FatiScan software. Translation efficiencies were graphically ranked in green (ΔmRNA_PB_/ΔmRNA_TOT_≥1) to red (ΔmRNA_PB_/ΔmRNA_TOT_≤1) and the most significant GO terms enriched along the ranking were annotated on the right with the adjusted p-values. (**C**) Distribution (expressed as a cumulative percentage) of the relative translation efficiencies in response to heat stress for mRNAs in the whole Arabidopsis genome (solid line), genes related with the GO term “translation” (dashed line) or genes related with the GO term “stress” (dotted line). K–S test was used to statistically examine the variations in the distributions, using the whole genome as reference. The corresponding p-values are indicated in the figure.

In order to validate the microarray data, qRT–PCR analyses were performed to assess the variations in the levels of 18 transcripts in total RNA (ΔmRNA_TOT_, Figure 3A) and polysome-associated populations (ΔmRNA_PB_, Figure 3B). Among the different genes included in the analysis, a similar number of candidates from the different categories established in Figure 4A were selected. In general, the qRT–PCR results showed a good correlation with the microarray data. Moreover, the translation efficiencies (ΔmRNA_PB_/ΔmRNA_TOT_) obtained by qRT-PCR were almost identical to the microarray data for all the transcripts evaluated (data not shown). Thus, the qRT–PCR analysis confirmed the absence of biases imposed by the methodology adopted and validate the microarray results.

### Heat stress mediates rapid changes in the translation efficiency of cohorts of mRNAs that are relevant for the stress response

In order to evaluate the extent of the translational regulation, the different analyzed mRNAs were classified according to their relative translation efficiency in response to heat stress (ΔmRNA_PB_/ΔmRNA_TOT_) (Figure 4A). The mRNAs were scattered following a normal distribution with an average relative translation efficiency of 0.564 (dotted black line), which corresponds, approximately, with the observed 50% decrease in polysome association. However, as above-stated, some transcripts were especially more resistant or sensitive to the general translational repression. Accordingly, mRNAs were divided in three groups. Class I (Figure 4A, red; n = 696) included mRNAs with a relative translation efficiency ≤1.5 fold the average translation efficiency of the distribution, which were especially sensitive to the general translation inhibition. In Class III (Figure 4A, green; n = 456), the mRNAs with a relative translation efficiency ≥1.5 fold the average translation efficiency of the distribution were included. These mRNAs were slightly resistant to the general translation inhibition observed upon heat stress. Class II (Figure 4A, blue; n = 14946) included the rest of the genes, which showed an average translation efficiency.

In order to explore whether selective translation regulates specific aspects of the response of plants to the high temperatures, the enrichment in Gene Ontology (GO) terms along the distribution was analyzed using FatiScan (Babelomics 4.3 suite) [40]. This software allowed to scan the ranked list of mRNAs according to the relative translation efficiency (ΔmRNA_PB_/ΔmRNA_TOT_) and to identify enriched GO categories in the top or the bottom of the list (Figure 4B). Similar translational regulation of genes related with specific biological processes was observed, pointing out the physiological relevance of this translational control. Especially resistant mRNAs (Figure 4B, green) were enriched, among others, for genes related to the stress response caused by: water deprivation (GO:0009414, 2.41E-8), cadmium ion (GO:0046686, 1.03E-7), salt (GO:0009651, 6.35E-7), nematode (GO:0009611, 3.15E-6) and wounding (GO:0009611, 3.49E-6). Protein phosphorylation (GO:0006468, 5.23E-7) and oxidation-reduction processes (GO:0055114, 1.17E-6) were also over-represented. In contrast, especially sensitive mRNAs (Figure 4B, red) were significantly enriched, among others, for metabolic functions related with translation (GO:0006412, 3.87E-30) and ribosome biogenesis (GO:0042254, 5.61E-13). Similar results were retrieved when the genes included in the 3 translational classes referred before were individually analyzed with GeneCodis [37], [38], [39] (data not shown). As expected, Class I was enriched in genes related to translation while Class III was enriched in genes related to stress response. In order to confirm whether these results could also apply for other genes involved in these categories, cumulative curves were plotted separately for mRNAs in the whole Arabidopsis (solid line) genome, for genes related with the GO term “translation” (dashed line) or for genes related with the GO term “stress” (pointed line), and the distribution of the relative translation efficiency of the corresponding mRNAs in each group was analyzed (Figure 4C). Under heat stress, the translation efficiency of genes related with “translation” is clearly reduced when compared with the whole Arabidopsis genome, while genes related with the “stress” response are more efficiently translated. The Kolmogorov-Smirnov (K–S) test, which was used to statistically examine the variations in the distributions, confirmed the significance of these data (p<0.001). Thus, mRNAs coding for components of the protein synthesis machinery, a highly energy consuming activity, are negatively regulated at the translational level in response to heat, while the translation of mRNAs encoding proteins that mediate the stress response is selectively activated.

**Figure 5:**
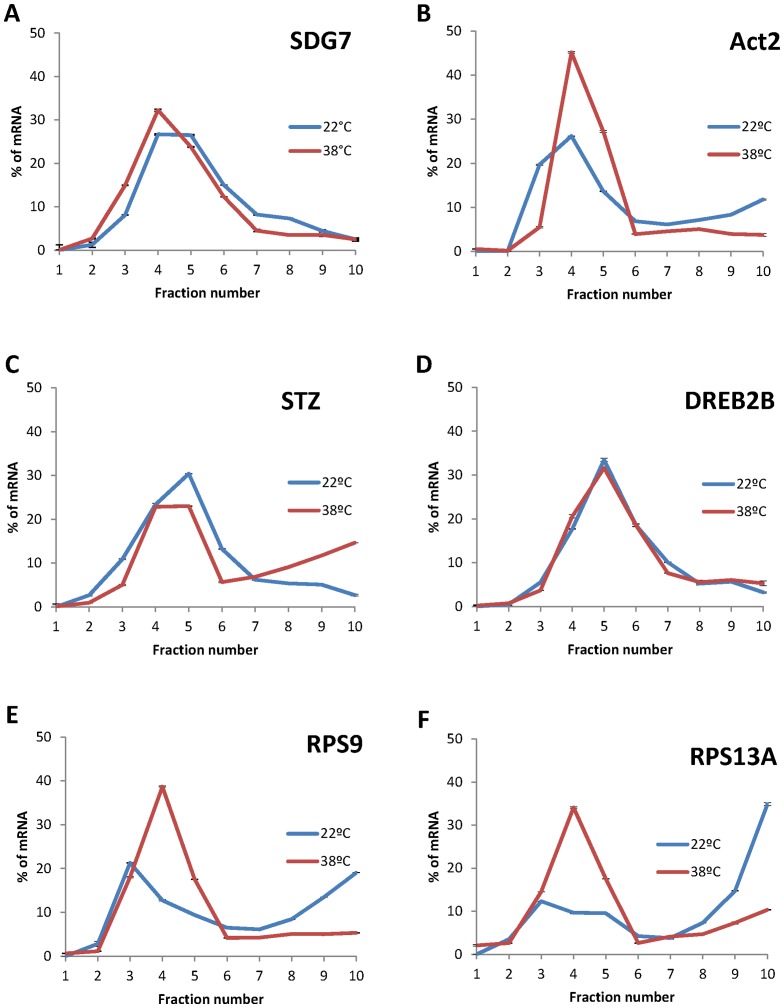
Heat-induced changes in polysome association for functionally relevant mRNAs. The distribution through the different fractions of a representative polysome gradient of the mRNAs coding for SDG7 (**A**), ACTIN2 (**B**), STZ/ZAT10 (**C**), DREB2B (**D**), the ribosomal protein S9 (**E**) and the 40′S ribosomal protein S13A (**F**) was analyzed by qRT-PCR, as specified in materials and methods. The percentage of the mRNA content within the different fractions at 22°C and 38°C are indicated in blue and red, respectively. Standard deviations were calculated from technical repeats.

In order to analyse the different patterns of mRNA polysome association imposed by high temperatures and further characterize the implication of the translational regulation in the modulation of key genes involved in the stress response and in ribosomal function, the heat-induced variations in distribution through the sucrose gradient of some individual mRNAs, which encode master regulators of the stress response, as STZ/ZAT10 (At1g27730) and DREB2B (At3g11020), or relevant ribosomal proteins, as the ribosomal protein S9 (At1g74970) and S13A (At4g00100), was evaluated by qRT-PCR (Figure 5 and S1). The relative amount of each mRNA in the different fractions was estimated from the Ct value and expressed as a percentage of the total mRNA in the gradient. The mRNA coding for the methyltransferase SDG7 (At2g44150) and ACTIN 2 (At3g18780) were used as controls. As shown in Figure 5A, the mRNA coding for SDG7 was displaced from the polysomal fractions in response to heat stress, in concordance with the average 50% reduction in polysome association observed in the ATH-1 arrays. The same binding pattern was observed for ACTIN2 (Figure 5B). However, according to the bioinformatic analysis, the mRNAs coding for the stress effector proteins STZ/ZAT10 and DREB2B (Figure 5C and 5D) were more actively associated or maintained its level of association to polysomes upon heat-stress, respectively. These results suggest that both were more efficiently translated than the mRNA bulk in these conditions. STZ/ZAT10 and DREB2B are two transcription factors [31], [45] that control the expression of numerous key stress-inducible genes. ZAT10 participates with DREB transcriptional factors in the activation of the stress signaling cascade, as they bind to cis-acting promoter sequences and control the expression, among others, of the cold regulated (COR) genes [46]. COR genes are considered key factors in the establishment of the stress response as they are coordinately transcribed and act in concert to enhance tolerance to different abiotic stress conditions in plants [47]. As, among other stress effectors, *STZ/ZAT10* and *DREB2B* are better engaged into polysomes under heat stress, these results highlight the relevance of the differential translation of master regulators in the proper establishment of the plant stress response to heat stress. On the opposite side, the mRNAs coding for the ribosomal protein S9 and the 40 S ribosomal protein S13A (Figure 5E and 5F) were significantly less associated to polysome in response to high temperatures and displaced to non-polysomal fractions, indicating that were strongly translationally repressed. All together, these results suggest that a selective regulation of the translation efficiency is fine-tuning the levels of gene expression to establish the stress response and to adapt plant metabolism in response to high temperatures.

### Differential translation in response to heat stress is partially defined by specific mRNA features

As inferred from the figures 2D and 4A, (green and red in these figures) a relevant group of mRNAs is differentially regulated at the translational level in response to high temperatures in Arabidopsis. In order to identify the individual features in the transcripts that could mediate their selective translation in this situation, correlations of their presence with the allocation of the mRNAs to the three translational functional groups were analyzed. Data sets for the cDNA, CDS, 5′-UTR and 3′-UTR sequences were retrieved from The Arabidopsis Information Resources (TAIR). The representative gene model was used as reference for each gene in the analysis. Only genes with complete information for all the different features analyzed were considered (n = 16098). The results are included in Table 1. Kolmogorov-Smirnov (K–S) test was used to validate the variations in the distribution of the individual features among the established groups, and statistically significant differences with a positive correlation with the relative translation efficiency were highlighted by an asterisk. In order to find out the characteristics that are representative of the whole set of mRNAs analyzed and discard those whose effect can be produced by a reduced group of particular genes, only features with relevant differences in the average value that displayed statistically different cumulative curves among the distinct three classes were considered relevant.

**Table 1:** Analysis of the representative features within the mRNAs in the different translation classes.

Feature analyzed	Class I	Class II	Class III
**Number of mRNAs**	696	14946	456
**cDNA length (nts)**	1204*	1641*	2323*
**CDS length (nts)**	857*	1279*	1981*
**5′-UTR length (nts)**	121	139	126
**5′-UTR G+C content (%)**	42.33*	37.61*	36.07*
**5′-UTR (1–10) G+C content (%)**	40.2*	36.3*	35.3*
**5′-UTR ΔG (kcal/mol)**	−21.13	−22.07	−18.63
**3′-UTR length (nts)**	226	223	216
**3′-UTR G+C content (%)**	32.29	31.64	31.02
**3′-UTR ΔG (kcal/mol)**	−40.69	−39.91	−37.49
**uAUG ≥1 (%)**	24.57	32.18	30.92
**uAUG ≥2 (%)**	11.21	15.84	14.47
**uAUG ≥3 (%)**	6.18	8.66	6.36
**uAUG ≥4 (%)**	3.59	5.09	3.73

Data sets for the indicated parameters of the representative gene models (n = 16098) were retrieved and analyzed as stated in Materials and Methods. Values are expressed as medians or as percentage when stated. Cumulative curves were plotted separately for all the features analyzed (data not shown). K–S test was used to validate the variations in the distribution of the individual features among the established groups, using Class II as reference. Statistically significant differences, with correlation with the relative translation efficiency, were highlighted by an asterisk (p<0.001).

**Figure 6:**
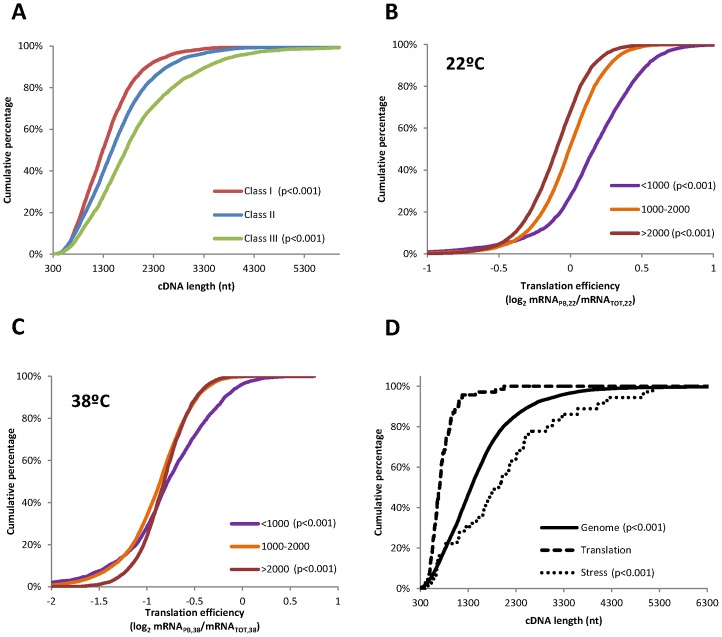
Heat selectively activates the translation of long mRNAs. (**A**) Study of the distribution of cDNA length among the three different translational classes described in Figure 4A. (**B–C**) Analyses of the distribution of the mRNA translation efficiencies according to the cDNA length. mRNAs were divided in three different categories according to their cDNA length (<1000 nts in purple, 1000–2000 nts in orange, and >2000 nts in brown) and the cumulative curves for the translation efficiencies within each cDNA length group were analyzed at 22°C or at 38°C, respectively. (**D**) Cumulative curves showing the distribution of cDNA length in the whole Arabidopsis genome (solid line, n = 16098), within the genes in class I related with translation (dashed line, n = 70) or within the genes in class III related with stress (pointed line, n = 36). In all cases, statistical differences were determined by K–S test, using Class II in (A), 1000–2000 nts cDNA length group in (B) and the whole genome in (C) as reference. The corresponding p-values are indicated.

Interestingly, translationally activated mRNAs (Class III) are 42% longer than the mean for the control group. In agreement with this positive correlation, translationally repressed mRNAs are, in average, 27% shorter than control mRNAs (Figure 6A). These results were unexpected, as previous studies in both yeast and human suggested a negative correlation of transcript length and translation efficiency [48], [49]. In order to further analyse this question, the influence of the cDNA length in the translation efficiency (mRNA_PB_/mRNA_TOT_) was evaluated separately at 22°C and 38°C. To do that, the mRNAs were divided in three different categories according to their cDNA length (<1000 nts in purple, 1000–2000 nts in orange, and >2000 nts in brown) and the distribution of the translation efficiency in each group was analyzed individually at both temperatures. In agreement with the previously published data, in control conditions the translation of shorter mRNAs is clearly favoured (Figure 6B). Thus, the effective translation of shorter mRNAs appears to be a conserved phenomenon in eukaryotic species. However, upon heat stress, long mRNAs are slightly better translated while short mRNAs are slightly worse translated (Figure 6C), explaining the results observed in Table 1. In order to assess if this mechanism could be also involved in the differential polysome loading of the genes related to stress and translation, cumulative curves were plotted separately to study the distribution of cDNA length in the whole Arabidopsis genome (solid line, n = 16098), within the genes in class I related with translation (dashed line, n = 70) or within the genes in class III related with stress (pointed line, n = 36) (Figure 6D). The repressed genes related with translation are clearly shorter (p<0.001) than the average in the genome, while the activated genes related with stress are clearly longer (p<0.001). These results indicate that cDNA length could be involved in the fine-tuned differential translational regulation in response to heat stress. Further analysis demonstrated that neither the 5′-UTR nor the 3′-UTR but the CDS length contribute to this translational control (Table 1).

**Figure 7:**
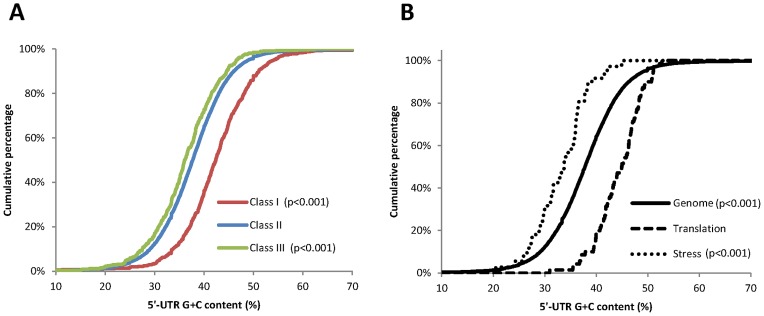
Heat selectively activates translation of transcripts with low 5′-UTR G+C content. Distribution analysis of the percentage of G+C content in the 5′-UTR of the mRNAs belonging to (**A**) the three translational classes described in Figure 4A or (**B**) within the whole Arabidopsis genome (solid line, n = 16098), within the genes in class I related with translation (dashed line, n = 70) or within the genes in class III related with stress (pointed line, n = 36). Statistical differences were determined by K–S test using class II or the whole genome as a reference in (**A**) and (**B**), respectively. P-values, to determine statistically significant differences, are also shown.

Features as the structure or the presence of uORFs have been previously proven to affect mRNA translatability [9], [13]. In order to analyze if the differential translation observed under heat stress could be modulated by these features, the G+C content, the ΔG and the presence of uORF within the genes assigned to the different translational classes were also tested. Regarding the influence of the 5′-UTR in the differential translation, the G+C content was found to be related with the translation efficiency in response to heat stress (Table 1). None of the other features analyzed in the 5′-UTR were significantly influencing the translational behaviour of the mRNAs (Table 1). Average G+C content of plant 5′-UTR (42,4%) significantly differs from that of human (60,8%), other mammals (59,5%) or fungi (40,9%) [50], [51] and these differences could be related with the mechanisms by which translational control is exerted in response to stress. Our data in Table 1 and in Figure 7A showed that translationally active mRNAs have a low G+C content (36% on average), while translationally repressed transcripts have a high G+C content (42% on average). This mechanism of selection also influences the differential polysome loading of genes related with stress and translation allocated to class III and I, respectively (Figure 7B). The repressed genes related with translation (dashed line) had clearly higher 5′-UTR G+C content (p<0.001) than the average in the genome (solid line), while the activated genes related with stress (pointed line) had clearly lower 5′-UTR G+C content (p<0.001). As stable RNA structures in close vicinity to the 5′end may control mRNA translation modulating the eIF4F and ribosome binding, we assessed whether the differences on translatability within the 3 functional classes correlated with the G+C content within the first 10 nucleotides in the 5′end of the UTRs. Although, in agreement with a recent report [52], the translationally activated mRNAs (class III) showed a reduction in the G+C content in this region, similar reductions were also observed for those genes in class II and for those in class I (Table 1). These data suggest that differences in translation efficiency among the three different groups may be not directly related to this particular mRNA feature.

Differential translation of mRNAs could be modulated by conserved sequences within the 5′-UTR that may enhance recruitment of the translational machinery directly or through the interaction with RNA-binding proteins [16]. Thus, to further evaluate whether mRNA cis- acting sequences in the 5′-UTR of the analyzed genes are associated with the regulation of the translational status in response to high temperatures, a search for overrepresented cis-elements within the 5′-UTR of the different classes was performed using Multiple Em for Motif Elicitacion (MEME-ChIP) [42]. The co-occurrence of the identified overrepresented elements in the rest of the groups was evaluated using Find Individual Motif Occurences (FIMO) [43]. Not evident significantly enriched consensus sequences were found. However, among the activated genes related with “stress”, different long A-T rich clusters were over-represented (data not shown), which is in agreement with the lower G+C content in the 5′-UTR of these genes.

All together, these data indicate that the cDNA length and the 5′-UTR G+C content may participate in the discrimination mechanisms involved in the differential translation of cohorts of transcripts in response to high temperatures. Further characterization of these mechanisms could allow gaining knowledge on how plants respond to heat stress, as the final expression of genes involved in the establishment of the stress response seems to be modulated by this translational control.

## Discussion

### Translation is drastically inhibited in response to heat stress in Arabidopsis seedlings

Different environmental stresses, such as high salinity, dehydratation, hypoxia or cadmium intoxication, impact on mRNA translation promoting re-adjustments in protein synthesis [6], [7], . Our results also confirm that translation plays an important role in the reprogramming of protein production under heat stress in Arabidopsis seedlings. Sucrose gradient fractionation demonstrates that a heat shock treatment of 38°C for 45 min results in a reduction of a 50% in the association of mRNAs to the polysomal fractions (Figure 1D). This inhibition is similar to the decline in polysomal binding observed in *Oryza sativa* cells after 10 min of heat shock at 41°C [28] or after 2 hours of severe hypoxia of Arabidopsis seedlings [6]. However, it doubles the inhibition obtained after mild heat shock (10 min at 37°C) [15] or following moderate water deficit in Arabidopsis cells and rosette leaves[12].

All these data demonstrate that the experimental conditions selected for this study cause a severe stress in terms of translation regulation and reinforce the previous evidence that this effect differs markedly depending on the cell type, severity and the duration of the stress.

### General and differential mRNA translation upon heat stress in Arabidopsis seedlings

At the individual mRNA level, heat shock exerts a general effect on the translation of the majority of mRNAs, reducing in an approximately 50% their loading to the ribosomes. However, not all the transcripts follow this general trend (Figures 2D and 4A). For a relevant group of them (almost the 10% of the genome), translation does not merely act as a non-selective regulator of gene expression, but it plays an active role in the selection of mRNAs for ribosome binding. In this sense, genes involved in cell survival under different abiotic threats are preferentially translated under heat stress conditions (Figure 4B and 4C). This selectively translated group of mRNAs is highly enriched in genes coding for effector proteins in response to water deprivation, cadmium ion, salt stress or wounding (Figure 4B). The selective translation of stress related proteins reinforces the importance of translation as a key mechanism to facilitate recovery from environmental insults. Interestingly, among the mRNAs preferentially translated upon heat stress, those coding for well characterized transcriptional regulators as STZ/ZAT10, which belongs to the ZPT2 related protein family [31], and DREB2B, a transcription factor from the DRE/CRT family [45], are included. The distribution of these mRNAs through the different fractions of the sucrose gradient further confirmed that heat stress promotes their association to the polysome fractions (Figures 5C and 5D). These transcriptional effectors control the expression of whole networks of stress response genes related to drought, high salinity and heat tolerance, including the expression of COR genes [46], [53]. As these transcription factors are considered master regulators of the transcriptional response to abiotic stresses [45], [47], a considerable effort has been made during years to uncover their transcriptional and post-translational regulation. Although more experimental evidence is needed to decipher the exact contribution of translation into the protein accumulation of these factors and to uncover the specific mechanisms that commit their mRNAs to preferential translation, the finding that these factors are targets of the translational control under heat stress adds another layer to their highly complex regulation. In addition, it makes a significant contribution to the knowledge of how plants launch specialized gene expression programs that promote stress protection and survival.

As part of the heat stress response, the translation efficiency of genes related to translation and ribosome biogenesis was preferentially reduced (Figures 4B–C and 5E–F), promoting energy saving and preventing the accumulation of proteins that might misfold as a consequence of elevated temperature [54]. Misfolded or unfolded proteins accumulate in the ER, triggering an unfolded protein response (UPR) that can lead to apoptosis or programmed cell death under acute or chronic stress conditions [55]. Similar reductions in the translation of ribosomal structural components were recently observed during the ER stress response in mammalian cells [56]. In this sense, the translational regulation of mRNAs involved in protein synthesis further confirms the role of translational reprogramming in mitigating the effect of heat on plant physiology. In addition, it establishes a negative loop of regulation by which the inhibition at the translational level of genes involved in translation will contribute to regulate tightly the protein synthesis blockage observed under heat stress.

Differential translation of mRNAs allocated to the similar functional categories was also found in response to other abiotic stresses in plants [6], [7], [8], [10], [14], [15], [28]. These results suggest the existence of common regulatory mechanisms or, alternatively, of different regulatory networks that converge on the differential translation of key cohorts of genes. In nature, heat stress usually threatens the fitness and productivity of plants in combination with other stress factors, such as water limitation and high UV irradiation. Thus, the activation under a specific challenge of genes that are common for the response to different threats may form part of the adaptation strategy of plants to cope with complex changes in environmental conditions.

### mRNA features that affect preferential translation under heat stress in plants

Two previous studies have described a reduction of mRNA polysome binding under heat stress conditions in plants [15], [28]. However, none of these studies have performed genome-wide analysis to evaluate the possible involvement of mRNA specific features within translation regulation. To address this important question, we have carried out a survey for representative features in those mRNA whose translation is preferentially enhanced or inhibited under heat stress in Arabidopsis seedlings. Among the different parameters tested, our results clearly demonstrate that the CDS length and the 5′-UTR G+C content highly influence the mRNA translation efficiency under heat stress (Figure 6A and 7A).

5′-UTR G+C content has been previously identified as a mechanism involved in the selective mRNA ribosome loading under mild dehydration stress, oxygen deprivation and darkness [7], [10], [13]. However, in some of these and similar studies, a preference for short mRNAs was previously described [7], [9], [10]. Our analysis plainly shows that mRNAs with higher translational efficiencies upon heat stress have longer cDNAs (Table 1). A likely explanation to this observation could be derived from the fact that long mRNAs with a high translational initiation rate could support the binding of larger number of ribosomes or, alternatively, it is possible that ribosomes remain attached to long mRNAs for longer under these particular stress conditions. Both effects could be reflected on an increase, not only in ribosome occupancy, but also in the ribosome density of the transcripts allocated in class III. In this regard, previous studies have shown that yeast under amino-acid starvation or Arabidopsis during morphogenesis could alter their translation patterns through the control of ribosome density [9], [57], [58]. Although, in this study, we have focused on ribosome occupancy, deciphering the relevance of ribosome density on the preferential translation of stress-related genes could contribute to understand how plants adapt to the environmental conditions.

### Heat stress regulation of translation initiation in plants

5′-UTR G+C content and cDNA length are two mRNA structural parameters that affect translation initiation. Indeed, a high G+C content in the 5′-UTR can greatly impair ribosome scanning and, depending on the position, may affect ribosome entry to the mRNA [59]. In addition, the length of an mRNA may affect the efficiency of the 5′-3′-end interaction needed for the mRNA circularization. The involvement of these two parameters in the selection mechanism for mRNA ribosome loading, along with the increase in monoribosome-bound mRNAs and ribosomal free subunits observed upon heat shock (Figure 1C), suggests that translation could be regulated at the initiation phase in Arabidopsis seedlings in response to high temperatures.

Translation is also mainly regulated at the initiation step in other eukaryotes [60], [61]. However, the mechanisms involved in this regulation differ considerably among species. These mechanisms range from the phosphorylation of different eIFs to the association of eIF4E with the 4E-BPs, and the regulation of the eIF4G by HSP27 association and insolubilization [62], [63], [64]. In plants, the mechanisms that regulate translation in response to heat stress remain largely unknown [16]. Indeed, although phosphorylation of plant eIF2*α* by GCN2 kinase modulates protein synthesis in response to different abiotic stresses, under our experimental conditions, heat stress does not lead to eIF2*α* phosphorylation (Figure S2), confirming previously published results in wheat [65]. Moreover, no homolog of the 4E-BPs has been found in the plant genomes available to date, questioning the existence of this conserved mechanism of regulation in the plant kingdom [16]. In wheat, dephosphorylation of eIF4B has been shown to correlate with the prompt inhibition of translation following heat shock [65]. This change may explain, in part, the negative correlation between translation efficiency and the 5′-UTR G+C content observed upon heat shock. Dephosphorylation of eIF4B under high temperatures may limit the eIF4A helicase activity. Under such conditions, those mRNAs with lower G+C content, and therefore with lower requirement for eIF4A activity, may be preferentially translated while those with higher base pairing may be translationally inhibited. Alternatively, base pairing and secondary structure can influence the binding of specific eIF4F isoforms [66] or of different eIFs, ribosome subunits or RNA binding proteins that may potentially affect translation of mRNAs with different G+C content in their 5′UTR. However, the participation of these proposed mechanisms in ultimately regulating the differential translation of mRNAs in response to heat stress should be examined. In other eukaryotes, multiple mechanisms operate simultaneously to assure the proper establishment of translation regulation under stress and the same situation may be possible in plants. Elucidating the factors that are involved in translation regulation is one of the important challenges that are remaining if we want to deepen our understanding of how plants respond to environmental stresses.

## Conclusions

**Figure 8:**
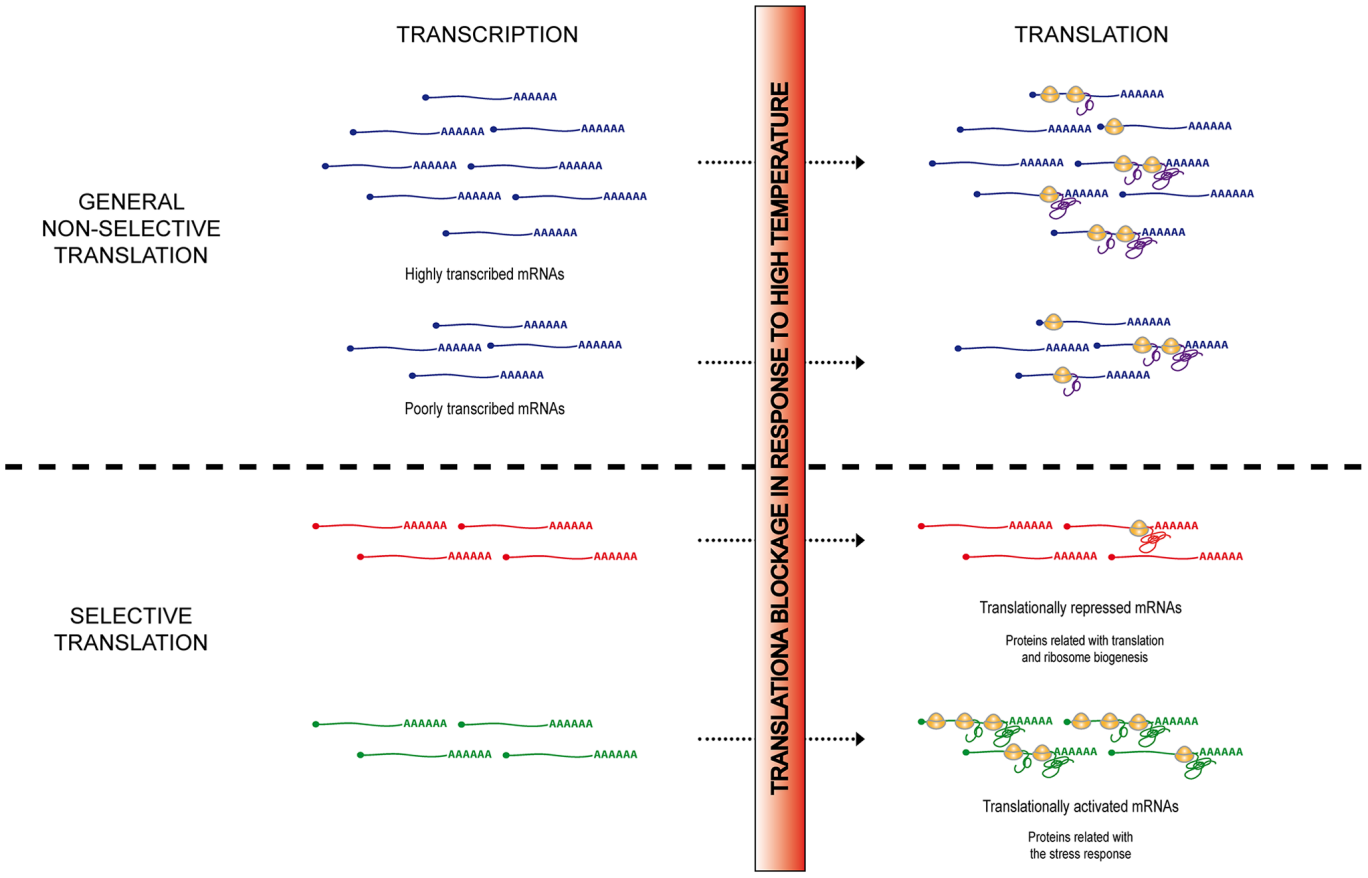
Model for mRNA translation regulation under heat stress in Arabidopsis seedlings. In response to high temperatures, translation is drastically inhibited. However, this translational blockage does not affect all the mRNAs equally. For the majority of the genes, a general and constant reduction in, approximately, a 50% in their loading to the ribosomes is observed (General non-selective translation). As selective ribosome loading is not exerted, the presence of these mRNAs into the polysomal fraction depends mainly on their abundance. Subsequently, for this group of genes, the final expression will be ultimately determined, although translational corrected, by the individual mRNA steady-state level. However, for a relevant group of mRNAs, selective ribosome binding is observed upon heat stress (Selective translation). Translationally activated mRNAs include genes involved in the establishment of the response to stress. In contrast, genes related to translation and ribosome biogenesis were translationally repressed. mRNAs form both differentially translated groups play a pivotal role in the maintenance of the cell homeostasis under stress conditions, suggesting an important role for the regulation of translation in the physiological response of plants to elevated temperatures. This model is focused on the regulation of transcription and translation under heat stress; however the possible involvement of other layers of regulation of gene expression in the final protein output should not be discarded.

In plants, as it is the case for all eukaryotes, gene expression reprogramming constitutes one of the major responses to abiotic stresses. This reprogramming is a highly complex process, as the accumulation of a specific protein results from a plethora of combinatorial processes, each of them finely regulated under environmental challenges. This mixture of processes covers from transcription to mRNA processing, mRNA transport and stability, translation or protein turnover; each participating but having a different weight in the final protein output. Although we already acknowledge that some of these processes (e.g. transcription) play a pivotal role in this protein reprogramming, however, we are only beginning to understand how translation is coordinated with the other processes to modulate protein expression under stress. In this report, we have used a high-throughput technology to evaluate the contribution of translational regulation in the final expression of plant genes under heat stress conditions. Our results clearly establish that general and differential translation exerts a wide regulation of gene expression in Arabidopsis seedlings upon heat stress. A model summarizing the heat stress mRNA translational regulation is shown in Figure 8. Furthermore, this report describes for the first time that mRNA features as G+C content and cDNA length seem to participate in the discrimination mechanism involved in the plant mRNA selective translation under high temperatures. Even more, it unravels important stress-regulatory genes as novel targets of translational control. This molecular knowledge gets us closer to understand the plants′ heat response and tolerance mechanisms and it may be used for engineering plants that can normally grow and reproduce under heat-stress conditions.

## Supporting Information

Figure S1 **Biological replicates of heat induced changes in polysome association for functionally relevant mRNAs.** Distribution of mRNAs through the different fractions was assayed as described for Figure 5.
(TIFF)

Figure S2 **Immunoblot analysis of eIF2α phosphorylation in response to high temperature.** Arabidopsis seedlings were untreated (−), treated with glyphosate for 2 h (Gly), an herbicide that promotes eIF2α phosphorylation [67], or incubated at 38°C for 45, 90 and 150 minutes. Phosphorylation of eIF2α was monitored using an antibody that specifically recognizes the eIF2α phosphorylated form at Ser51 (upper panel). HSP101 and ACTIN levels were assayed as control of the heat shock treatment (middle panel) and as loading control (lower panel), respectively.
(TIFF)

Table S1 **Primer sequence of the genes used in this study for qRT-PCR analyses.**
(PDF)
