# Supplementary figures and images for: Analysis of Genome-Wide Changes in the Translatome of Arabidopsis Seedlings Subjected to Heat Stress
Source: PLoS One. 2013 Aug 19;8(8):e71425. doi: 10.1371/journal.pone.0071425 (PMC3747205; doi:10.1371/journal.pone.0071425)

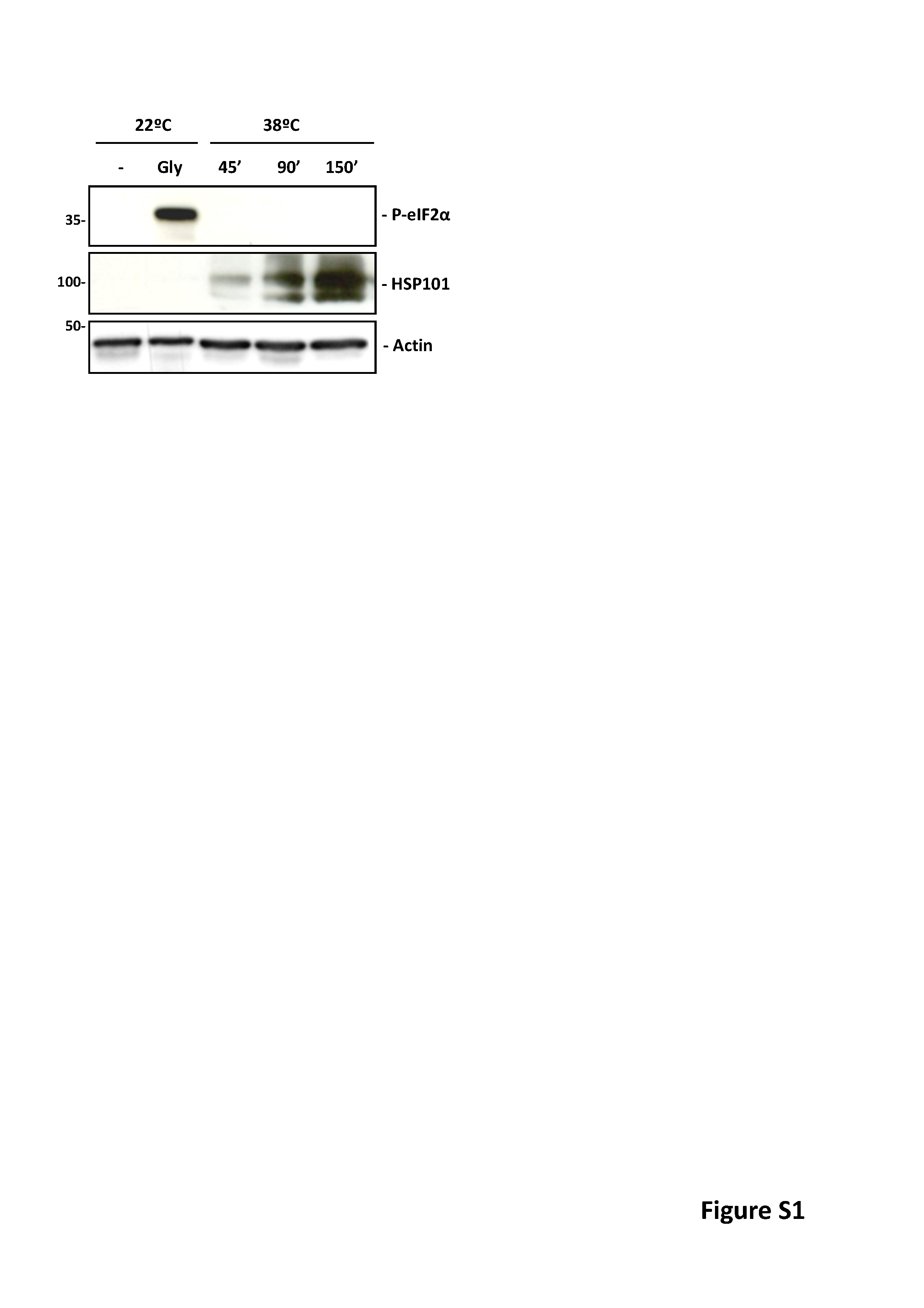

Supplement: Figure S1 — Biological replicates of heat induced changes in polysome association for functionally relevant mRNAs. Distribution of mRNAs through the different fractions was assayed as described for Figure 5. (TIFF) [file pone.0071425.s001.tiff]

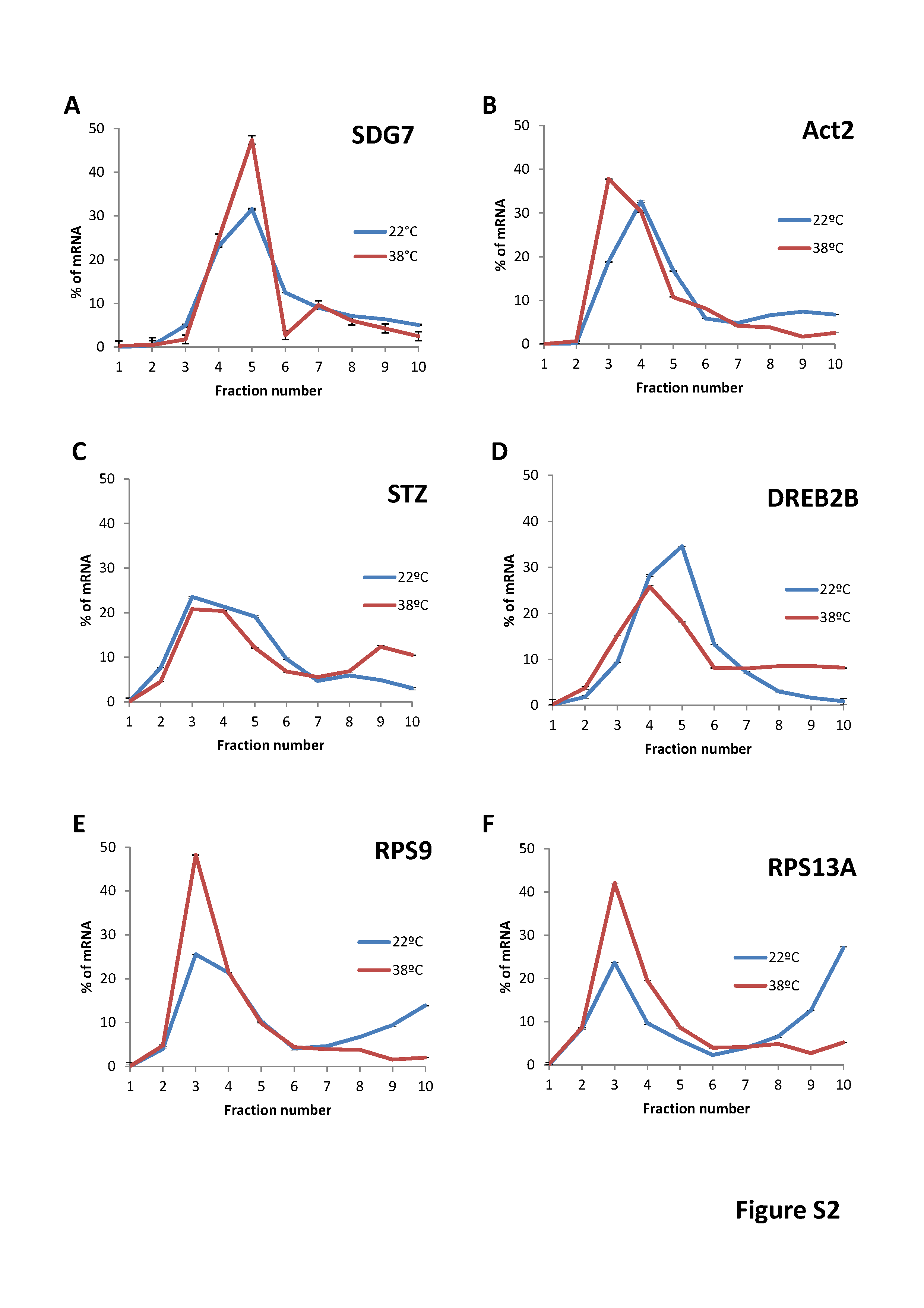

Supplement: Figure S2 — Immunoblot analysis of eIF2α phosphorylation in response to high temperature. Arabidopsis seedlings were untreated (−), treated with glyphosate for 2 h (Gly), an herbicide that promotes eIF2α phosphorylation [67], or incubated at 38°C for 45, 90 and 150 minutes. Phosphorylation of eIF2α was monitored using an antibody that specifically recognizes the eIF2α phosphorylated form at Ser51 (upper panel). HSP101 and ACTIN levels were assayed as control of the heat shock treatment (middle panel) and as loading control (lower panel), respectively. (TIFF) [file pone.0071425.s002.tiff]
