# Supplementary material for: Analysis of Genome-Wide Changes in the Translatome of Arabidopsis Seedlings Subjected to Heat Stress
Source: PLoS One. 2013 Aug 19;8(8):e71425. doi: 10.1371/journal.pone.0071425 (PMC3747205; doi:10.1371/journal.pone.0071425)
Supplement: Table S1 — Primer sequence of the genes used in this study for qRT-PCR analyses. (PDF) [file pone.0071425.s003.pdf]

Table S1. Primer sequence of the genes used in this study for the analysis by qRT-PCR.

Gen (TAIR ID)	Forward primer (5'-3')	Reverse primer (5'-3')
**At1g12240**	GACACGAGGTTCGATCATTATGCTG	GACCGACCTCGAGATCAAATTGC
**At1g20630**	CGCCATGCCGAAAAATACCC	CTTGCCTGTCTGAATCCCAGGAC
**At1g27730**	GCTTCTCCGATTCCTCCTTT	TCTTGTCGCAGACGCTACAC
**At1g52890**	GTTCCACGGTCTTGCGGATAC	GCTCAACATTACCGGCAAAACTAG
**At1g74970**	TTCTTCGTTCAGCGACAATG	AGATACCGTGGCGGTGATAG
**At2g21580**	GCCAAAGAAGGATAAGGTTCCACCA	CCTCGCAAGAGACCCATTAATCCTC
**At2g23170**	CGGTTTGGTCTCAAGGGATTCAC	GTCGGTCCATGTCTTCATCAATGG
**At2g42740**	CGTTTCGTTCTCCGTCTCTC	AAAACCTTGGAGGCTCTGGT
**At3g03780**	TGCCTCCTGGATTCAGCTTGATGAG	GCCTTGCGCTAACTTCAGTTGTACG
**At3g11020**	TCGAGATGAAGCGGATGCAAATCA	TGAATGAACCTGGTCCCCATCAGA
**At3g17465**	GGATCATCGCGGTTAAGTGT	GCACACCTTGTGCTCTGAAA
**At3g18740**	GTCCCATGAAGGAATCAACAGCAG	CATTGTTGCCATTGTAGCGATGAAC
**At3g18780**	ACCTTGCTGGACGTGACCTTACTGAT	GTTGTCTCGTGGATTCCAGCAGCTT
**At3g28900**	CCGCATCGTCAAAACTCCTGGTG	CGATCCTCTCCCTAACAGCAACTCC
**At4g00100**	CATGGGTCGTATGCACAGTC	TCTTGACCTGAGGGATACCG
**At4g25490**	GCATGTCTCAACTTCGCTGA	ATCGTCTCCTCCATGTCCAG
**At4g33950**	TTTGCCGATTATGCACGATA	TGGCTAAATGGGTTGGTGTT
**At4g34000**	AACCGTTCTCAACCTGCAAC	TTGGAGTCAGATCAGGTGACAT
**At4g38620**	TCCCGGCAATAAGCGACCATTTG	TCACCGAGGAAGAAGACGAACTCA
**At5g02940**	AGAACCTTGGCACGGTATTTGTGG	GGCAGCCCTTTCAAACGATTTTGTC
**At5g04620**	TGTTCGACGGTTTGTGTCAATGG	TGATTCAGCGCATCAGAAAAGATGG
**At5g15960**	ACCAACAAGAATGCCTTCCA	CCGCATCCGATACACTCTTT
**At5g52310**	TCACCGGAACTTTCTCATCC	TGCTCTGTTTTGGCTCCTCT
